# Genome-wide association study (GWAS) for body weights of sussex cattle (*Bos taurus*) in South Africa

**DOI:** 10.1016/j.heliyon.2024.e39540

**Published:** 2024-10-18

**Authors:** Lubabalo Bila, Widya Pintaka Bayu Putra, Dikeledi Petunia Malatji, Yandisiwe Patience Sanarana, Thobela Louis Tyasi

**Affiliations:** a Department of Animal Production, Potchefstroom College of Agriculture, Potchefstroom, 2520, South Africa; b Department of Agriculture and Animal Health, College of Agriculture and Environmental Sciences, University of South Africa, Florida, 1710, South Africa; c Research Center for Applied Zoology, National Research and Innovation Agency (BRIN), Bogor, 16911, Indonesia; d Agricultural Research Council - Animal Production, Irene, Pretoria, 0062, South Africa; e Department of Agricultural Economics and Animal Production, School of Agricultural and Environmental Sciences, University of Limpopo, Sovenga, 0727, South Africa

**Keywords:** Body weights, Candidate genes, GWAS, SNP markers, Sussex cattle

## Abstract

Sussex cattle (*Bos taurus*) is one of beef cattle breeds in South Africa and it is possible to improve their genetic potential through genomic selection. The aim of this study was carried out to implement Genome-wide Association Study (GWAS) for determining candidate genes of body weights at birth, weaning and yearling stages in Sussex cattle. A total of 96 heads of Sussex cattle consisting of 45 females and 51 males were used for GWAS with Bovine 100K SNP BeadChip (Illumina, USA). The results showed that three SNP markers of BovineHD1000018504 (SNP1), BovineHD100008645 (SNP2) and BovineHD2500008434 (SNP3) were detected as the potential genetic markers for body weight of animals under this study. Among the three identified SNPs, SNP1 was not located in any gene region while SNP2 and SNP3 were located in the region of *LOC100335918* (intron 3) and *SCLT1* (intron10) genes, respectively. Therefore, both the identified SNPs had the relationship with the body weight at different ages of the Sussex cattle based on the Manhattan plot graphic *i.e.* birth weight (BW) for SNP2 and weaning weight (WW) for SNP3. In conclusion, a significant association was observed between SNP3 and WW of South African Sussex cattle. Nonetheless, there are no association between SNP2 and BW of Sussex cattle.

## 1 Introduction

The Sussex cattle (*Bos taurus*) is one of the beef cattle breeds found in South Africa (SA) and it is possible to improve their genetic potential through genomic selection. Sussex beef cattle can produce birth, weaning and yearling weights of 31.48, 250.62 and 404.48 kg, respectively [1]. Sussex cattle selection program focusing mainly on increasing production efficiency of growth traits is essential as the potential beef cattle of SA. Body weight is one of the most economic important growth traits however farmers and researchers find it difficult to measure [1]. The genome-wide association study (GWAS) is a great tool for recognising loci and individual polymorphisms correlated with economic important traits in numerous species of animals [2]. In the recent years, molecular biology, genetics, and bioinformatics have made great strides, which has strongly advanced animal genomics research. Moreover, GWAS provides valuable information to improve the understanding on the genetics of complex traits that are difficult to measure in cattle. In cattle, GWAS can find loci that are associated with body weight (BW), carcass traits and meat quality traits in Nellore cattle [[3], [4], [5]] and fatty acid composition in Simmental and Wagyu cattle of China[6,7] In GWAS, the information of thousands of single nucleotide polymorphisms (SNPs) distributed uniformly throughout the genome is used together with the animals phenotypes and pedigree information to perform association analysis and identify genes involved in the control of traits of economic importance [8]. Buzanskas et al. [9] reported that growth traits are often used for selection and are those measurements of body weight that can be recorded from birth, weaning and throughout the life of the animal. Generally, heritability and genetic correlation coefficients for growth traits are medium to high [10]. Hence selection based on genetic merit for these traits applied over multiple generations has been effective to increase the postnatal growth in several beef cattle breeds. Unfortunately, studies to determine the candidate gene for body weight and productive traits on South Aftican Sussex cattle with GWAS are still limited [3,4]. Hartati et al. [11] showed that GWAS has identified the PLAG1 gene as the candidate gene for the calf birth weight of Ongole grade cattle. However, to the greatest of our knowledge, there is no literature documented yet on genome-wide association study for the body weight of Sussex cattle (*Bos taurus*) in South Africa. Hence, the objective of this research was to implement the genome-wide association study for determining candidate genes of body weight in Sussex cattle. The findings of this research might be important for implementing future genetic improvement programs.

## 2 Materials and methods

### Ethics approval

The experimental procedures were conducted following the University of South Africa (UNISA) Ethics code for the use of live animals in research with ethics reference number: 2022/CAES_AREC/171.

### 2.1 Animals, research site and management

A total of 96 animals comprising 45 females and 51 males were used for the study. The animals were kept at the Huntersvlei, also known as Rhys Evans Group farm (RE) in Free State Province, South Africa. The farm is located in Viljoenskroon, Fezile Dabi municipality. The research site has an astronomical location at latitude −27°20′21.05″ N and longitude 27°43′9.38 E with the elevation of 1471 m asl; air temperature of ±30.4 °C; relative hummidity of 57–89 % and rainfall of ±650 mm/year. All the animals were kept on the traditional pasture system which allows animals to freely graze during the day and afternoon with the *ad libitum* water and regular health inspection.

### 2.2 Body weights

The data records of birth weight (BW), weaning weight (WW) and yearling weight (YW) were collected from each animal. The animal weighing was done once at birth, weaning and yearling stages using a digital weighing scale (Tal-Tec, South Africa). Two groups of animals *i.e.* weaning (6–8 months of age) and yearling (12–15 months of age) were managed in this study. Therefore, the weaning (55 heads) and yearling (41 heads) groups were managed for the genomic analysis separately. The average body weight in animals under study is presented in Table 1.

**Table 1:** The average of body weight (kg) in Sussex cattle.

Sex	Period (N)		
	Birth	Weaning	Yearling
Male	40.41 ± 4.44 (51)	264.24 ± 38.17 (21)	437.17 ± 64.83 (30)
Female	37.48 ± 5.73 (27)	243.41 ± 39.94 (34)	326.36 ± 28.82 (11)

Pool	39.40 ± 5.08 (78)	251.36 ± 40.24 (55)	407.44 ± 75.67 (41)

N: number of animal.

### 2.3 Genotyping

The hair samples were collected from each animal to obtain the genomic DNA material. The DNA was extracted using the Zymo Research Quick-DNA™ Miniprep Plus Kit (Zymo Research, USA) following the manufacturer's protocol and quantified using the Qubit dsDNA HS assay kit (Thermo Fisher Scientific). The DNA samples were genotyped using the Illumina Bovine 100K SNP BeadChip panel consisting of 95,256 markers at NEOGEN laboratory services in Lincoln, Nebraska (Illumina, USA).

### 2.4 Filtering and selecting of SNP markers

Filtering and selecting of SNP Markers were performed using TASSEL 5.0 software [12]. Genotype samples with less than 90 % call rates were removed from the analysis. Non-autosomal SNPs including those with unknown positions were removed. SNPs with minor allele frequency value (MAF<0.05); call rate (<95 %) and Hardy Weinberg Equilibrium (p-value <10^−6^) were not considered further for the analysis. A total of 82,568 autosomal SNP markers (BTA1 - BTA29) were used for the analysis to obtain the candidate genes. In addition, the Quantile-Quantile (Q-Q) and Manhattan plots were computed with GLM statistics method to select the associated traits and the best SNP markers, respectively. In this study, a threshold value of -Log_10_ P^5^ was adopted to select the best candidate SNP markers that can be associated with body weight of the animal.

### 2.5 Gene annotation and data analysis

Detection of the SNP marker position was assessed according to the *Bos taurus* genome (Btau_5.0.1: GCF_000003205.7) that available on GenBank database (https://www.ncbi.nlm.nih.gov). The genetic diversity parameters such as genotype frequency, allele frequency, observed heterozygosity (H_o_), expected heterozygosity (H_e_), polymorphic informative content (PIC), number of effective allele (n_e_) and Chi-square (χ^2^) values were estimated in the selected SNP [[13], [14], [15], [16]]. The analysis of variance (ANOVA) was computed to determine the association between SNP marker and body weight of animal using mathematical formula from Falconer and Mackay [17] as follows:

$$Y_{\text{ijk}}=\mu +\alpha_{i}+\beta_{j}+{Ɛ}_{\text{ijk}}$$

Where: Y_ijk_ is the observed trait; μ is the common mean; α_i_ is the effect of sex; β_j_ is the effect of genotype and, Ɛ_ijk_ is the experimental error.

## 3 Results

**Fig. 1:**
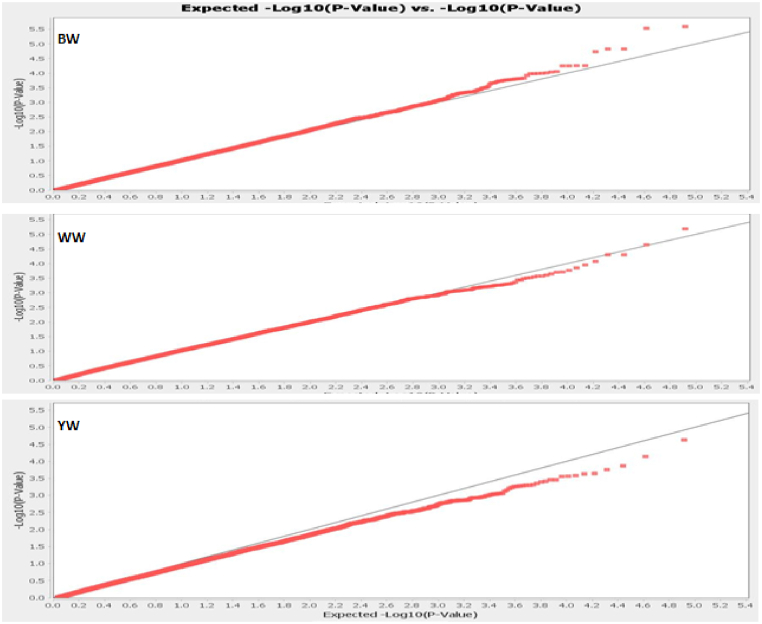
Quantile-Quantile (Q–Q) plot of the selected p value (-Log_10_ P^5^) for individual SNP markers. The line indicates the expected threshold values when confirming the null hypothesis of the absence of association. BW = body weight; WW = weaning weight; YW = yearling weight; ■ = SNP marker; ─ = expected threshold line.

**Fig. 2:**
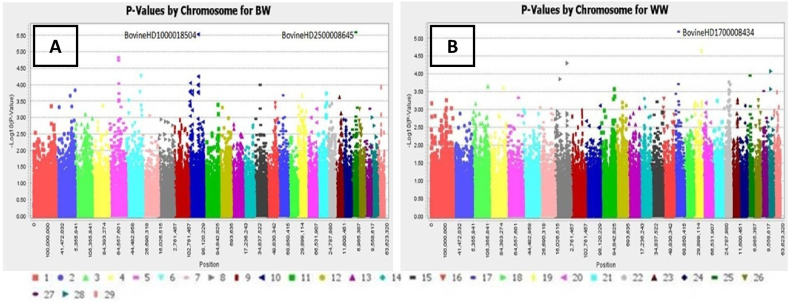
Manhattan plots for the GWAS in Sussex cattle from chromosmes 1–29 (A) Birth weight (BW) and (B) Weaning weight.

Generally, the BW, WW and YW in males were higher than the body weight in females animals however not statistically different as shown in Table 1. Moreover, the pool animals had the BW, WW and YW of 39.40 ± 5.08 kg, 251.36 ± 40.24 kg, and 407.44 ± 75.67 kg, respectively. In addition, the Q-Q plot indicates that BW and WW traits are spread above the threshold line while the YW trait is spread under the threshold line (Fig. 1). Fig. 2 (A and B) presents the visualization results of the location of statistically significant polymorphic sites across 29 chromosomes for body weight at birth (Fig. 2A) and yearling weight (Fig. 2B) of South African Sussex cattle. Moreover, Based on the Manhattan plot, two SNP markers significantly associated with BW trait were detected on BTA 10 (BovineHD1000018504) and 25 (BovineHD2500008645) (Fig. 2A). There results showed only one SNP significantly associated with WW trait and was identified on BTA 17 (BovineHD1700008434). All the SNPs detected had the p and MAF values < 1.00E-6 and >0.20 (Table 2). SNPs markers detected for the BW were located at *LOC100335918* (Intron 3) and *SCLT1* (Intron 10) genes region at positions 30,675,481 - 31,534,023 and 29,224,895 - 29,453,181, respectively as shown in Table 3. The one SNP in position 63,863,849 detected for WW was not located at any gene region. Subsequently, the PIC value in the *LOC100335918* and *SCLT1* genes were 0.21 (moderate) and 0.37 (high), respectively as shown in Table 4. Despite this, the genetic diversity in both genes were shown under the genetic equilibrium. Nonetheless, the polymorphism of *LOC100335918* gene was not significantly associated with BW trait. While, the polymorphism of *SCLT1* gene was significantly associated with WW trait (Table 5). Moreover, the AA genotype of *SCLT1* gene carried the inferior trait.

**Table 2:** Detection of potential SNP markers for the growth traits in Sussex cattle.

SNP Maker	Associated trait	BTA	P-value	MAF
BovineHD1000018504	BW	10	2.91E-6	0.21
BovineHD2500008645	BW	25	2.52E-6	0.22
BovineHD1700008434	WW	17	6.49E-6	0.29

BW: birth weight; WW: weaning weight; MAF: minimum allele frequency; BTA: *Bos taurus* autosomal chromosome.

**Table 3:** Detection of the candidate genes according to selected SNP markers^a^.

SNP Marker	Position	Gene	Region	Gene position
BovineHD1000018504	63,863,849	–	–	–
BovineHD2500008645	31,170,620	*LOC100335918*	Intron 3	30,675,481 - 31,534,023
BovineHD1700008434	29,339,304	*SCLT1*	Intron 10	29,224,895 - 29,453,181

a Assembly:Btau_5.0.1 (GCF_000003205.7).

**Table 4:** Genetic diversity in the candidate genes for growth traits of Sussex cattle.

Gene	N	Genotype frequency			Allele frequency		H_o_	H_e_	n_e_	PIC	χ^2^
*LOC100335918*	78	TT (0.73)	TC (0.27)	TT (0.00)	T (0.87)	C (0.13)	0.27	0.23	1.30	0.21	1.89^a^
*SCLT1*	55	AA (0.22)	AC (0.49)	CC (0.29)	A (0.46)	C (0.54)	0.49	0.50	1.99	0.37	0.01^a^

N: number of observation; H_o_: observed heterozygosity; H_e_: expected heterozygosity; n_e_: number of effective allele; PIC: polymorphic informative content; χ^2^: Chi-square value.

a Under in a genetic equilibrium.

**Table 5:** Association between gene polymorphisms and growth traits of Sussex cattle.

Gene polymorphism	N	Growth traits (kg)	
		Birth weight	Weaning weight
*LOC100335918*			
TT genotype	57	38.05 ± 4.84	–
TC genotype	21	43.24 ± 3.55	–
*SCLT1*			
AA genotype	12	–	214.00 ± 38.24^a^
AC genotype	27	–	254.48 ± 32.89^b^
CC genotype	16	–	274.12 ± 34.56^b^

N: number of observation.

## 4 Discussion

Schoeman [18] reported that Sussex cattle had the BW and WW of 36 kg and 214 kg, respectively. In addition, a recent study conducted by Bila et al. [1] reported the WW trait of the Sussex cattle to be 269.27 ± 37.59 kg (males) and 236.23 ± 39.55 kg (females). A number of studies identified the SNP Makers with the P = <1.0E-6 for body weight of cattle [3,[19], [20], [21], [22]]. Previous studies obtained the candidate gene at BTA10 region and associated it with growth and feed efficiency traits of European cattle *i.e. TMEM62, HERC1, TCF12, TEX9, UNC13C, GALNT16,* and *CEP128* genes [23] and average daily gain (ADG) of Nellore cattle *i.e. RAD51B* gene [20]. In addition, BTA17 had the candidate genes for growth and feed efficiency traits of European cattle *i.e. TMEM132C* and *MSI1* genes [23] and body weight gain of *Bos indicus* cattle *i.e. RPL6* gene [3]. Martinez et al. [24] reported that BTA17 had five candidate genes (not described) associated with WW of Colombian Brahman cattle. The body weight of cattle can be influenced by breed health status, genetic makeup, nutrition and climate factors. Hence, it can be suggested that BW and WW able to improve with the molecular selection tools. Despite this, Hartati and Putra [2] obtained an intergenic SNP marker at BTA17 *i.e.* ARS-BFGL-NGS-78232 that are significantly associated with slaughter weight and carcass weight of Sumba Ongole bulls (*Bos indicus*). Furtheremore, BTA25 had the candidate genes for growth and feed efficiency traits of European cattle *i.e.NDE1* and *TPST1* genes [23] and body weight at 420 days of age in Canchim beef cattle *i.e. XYLT1* gene [9]. A *LOC100335918* gene is located at BTA25 along 858,543 bp with five exonic regions (GenBank: NC_007326.6) while *SCLT1* (*Sodium channel clathrin linker 1*) gene is located at BTA17 [24] along 228,287 bp with twenty-two (22) of exonic regions (GenBank: NC_007315.6). The *LOC100335918* or *Autism susceptibility gene 2 protein-like* (*AUTS2*) is important for neurological development in human [25]. Thus, the *SCLT1* gene encoded protein functions to link clathrin to the sodium channel protein type 10 subunit alpha protein in rat [26]. Both *LOC100335918* and *SCLT1* genes had the MAF value of more than 0.20. The MAF value can be classified into rare (<0.05), intermediate (0.05–0.25), and >0.25 for highly polymorphic [27,28]. Furthermore, the PIC value in the present study indicated that the genetic diversity of *LOC100335918* and *SCLT1* genes of Sussex cattle were in a category of moderate and high, respectively. Nei & Kumar [15] stated that the PIC value can be classified into low (<0.10), moderate (0.10–0.30) and high (>0.30). A genomic region at 79.48 Mb of BTA3 was associated with BW in Blanco Orejine (BON) cattle, containing two candidate genes, the leptin receptor (LEPR) and leptin receptor overlapping transcript (LEPROT). These two genes plays a significant role in regulating body energy homeostasis and metabolism [29] and are involved in the control of the growth hormone [30], which may constitute a molecular link between nutritional signals and the actions of GH in body growth and metabolism [31]. The genetic diversity in the *LOC100335918* and *SCLT1* genes of Sussex cattle were described under the genetic equilibrium. In livestock, the genetic diversity can be influenced by selection, migration, inbreeding and crossbreeding factors [16]. However, the significance association showed between the *SCLT1* genes polymorphism and WW trait with the AC as superior genotype. There are no previous studies reported on the effect of *bovine SCLT1* gene polymorphism to economic important traits. However, mutation in the human *SCLT1* gene causing the genetic disease of oral-facial-digital (OFD) syndrome [32,33].

## 5 Conclusion

Two candidate genes of *LOC100335918* (BovineHD2500008645) and *SCLT1* (BovineHD1700008434) were detected as the potential genetic markers for BW and WW traits of Sussex cattle, respectively. However, the *SCLT1* gene had the higher PIC value than *LOC100335918* gene (0.37 vs 0.21). Unfortunately, there are absence of significant SNP markers for YW trait of Sussex cattle based on Q-Q and Manhattan plots. Hence, the in-depth study with standardized body weight is important to obtain the genetic markers accurately.

## CRediT authorship contribution statement

**Lubabalo Bila:** Writing – original draft, Visualization, Validation, Software, Methodology, Investigation, Funding acquisition, Formal analysis, Data curation, Conceptualization. **Widya Pintaka Bayu Putra:** Writing – original draft, Software, Methodology, Formal analysis, Data curation, Conceptualization. **Dikeledi Petunia Malatji:** Writing – review & editing, Visualization, Validation, Supervision, Project administration, Methodology, Investigation, Formal analysis, Data curation, Conceptualization. **Yandisiwe Patience Sanarana:** Writing – review & editing, Visualization, Validation, Methodology, Formal analysis, Data curation, Conceptualization. **Thobela Louis Tyasi:** Writing – review & editing, Visualization, Validation, Supervision, Software, Resources, Project administration, Methodology, Investigation, Formal analysis, Data curation, Conceptualization.

## Data availability

All data generated during this study are available through a request to the corresponding author.

## Funding information

The authors would like to thank the 10.13039/501100001321National Research Foundation (NRF) reference number (MND210415594902) for its financial support.

## Declaration of competing interest

The authors declare that they have no known competing financial interests or personal relationships that could have appeared to influence the work reported in this paper.
